# Phosphorylated Cotton Cellulose as a Matrix for Generating Chlorine Dioxide

**DOI:** 10.3390/polym15040967

**Published:** 2023-02-15

**Authors:** Anfisa Kazberova, Roman Solovov, Verbina Orlichenia

**Affiliations:** Frumkin Institute of Physical Chemistry and Electrochemistry of the Russian Academy of Sciences, 40 Obruchev Street, 117342 Moscow, Russia

**Keywords:** phosphorylated cotton cellulose fabric, chlorine dioxide, sodium chlorite, UV treatment, tensile strength

## Abstract

Currently, developing disinfectant materials is of utmost importance. A significant advantage of our fabric is its reusability. The disinfectants based on a natural polymer of cellulose have been barely investigated. Our work presents a modified cellulose material, and the data obtained for the first time on the chlorine dioxide generation process when treating the material with a sodium chlorite alcohol solution. A method of applying NaClO_2_ onto the fabric by impregnating it with a solution sprayed by an aerosol generator is proposed. This kind of fabric is capable of withstanding multiple usages after pre-washing and rinsing. The lowest alcohols—methanol, ethanol and isopropanol—are proposed as optimal solvents. It was shown that the phosphorylated cotton cellulose fabric impregnated with this solution generates chlorine dioxide during the first 25–35 min. Neither humidity nor expedites improve the process of releasing the chlorine dioxide, but high moisture content in the air causes the complete absorption of ClO_2_ by microdrops and its removal from the gas environment. A promising technique for removing the excess ClO_2_ by the means of UV treatment is proposed: after 15 min of treating ClO_2_ in the gas phase, it disappears entirely. These materials could be used as disinfectants in different industries, such as food and industrial manufacturing.

## 1. Introduction

One of the development directions of modern polymer (both synthetic and natural) chemistry is the modification of polymers to obtain materials with the required properties. Cellulose fiber has several advantages in comparison to synthetic fiber: higher hygroscopy, higher thermal stability and better hygienic qualities [1]. However, there are several significant drawbacks: flammability, a tendency to wrinkle, low resistance to microorganism influence and low elasticity. Despite the aforementioned drawbacks, cellulose is the most widespread biopolymer, which makes it a rather important subject of research. The application fields of cellulose are expanding every year, due to its almost inexhaustible supply if used rationally.

Taking into consideration the current epidemiological situation in the world, developing systems and materials that allow effectively disinfecting the air indoors and air space around people is crucial and relevant. We propose to study antibacterial fabric for air disinfecting based on cotton cellulose fabric that was phosphorylated in advance, with sodium chlorite permeated on the material surface. During the interaction, an antimicrobial agent, chlorine dioxide, is released, which disinfects the air effectively by acceptable methods. Chlorine dioxide is recognized as one of the promising oxidizing agents of a wide spectrum that is effective against a large number of microorganisms [2]. However, the problem of developing the optimal technology for releasing its gas form and method of application is still unsolved.

Chlorine dioxide ClO_2_ acts as an oxidizing agent and reacts with the cellular components, including the cell membrane of microbes [3]. During oxidation, molecular bonds are broken, the exchange process is disrupted, which leads to the death of the organism as a result of cell destruction. The oxidation of proteins disrupts the enzymatic function of microorganisms, which causes the rapid destruction of bacteria. This oxidative attack prevents the mutation of microorganisms into a form resistant to the dioxide [4].

Unlike non-oxidizing disinfectants, chlorine dioxide kills microorganisms even when they are inactive. Therefore, the concentration of chlorine dioxide required for the effective destruction of microorganisms is lower than the concentration of non-oxidizing disinfectants.

Chlorine dioxide plays an important role as a part of various disinfectants in production facilities, especially in water purification zones and cooking zones [5].

The importance of its disinfecting properties was further enhanced during the outbreak of *Bacillus anthracis* in 2001. Fumigation with vaporous hydrogen peroxide and ClO_2_ was used to destroy *B. anthracis* spores. ClO_2_ has been proven to be a powerful disinfectant for preventing outbreaks of infectious diseases [6]. The study [7] showed that due to the interaction between ClO_2_ and biological thiol-containing groups of amino acids, bacteria cannot develop resistance to ClO_2_. In the study [8] with human skin keratinocytes, it was shown that ClO_2_-based disinfectants are effective in destroying *B. anthracis* spores in solutions and are less toxic than sodium hypochlorite. It is reported that ClO_2_, when used as a disinfectant on surfaces, exhibits antimicrobial properties against various types of microbes, even in humid environments. The concentration of ClO_2_ 700–1100 ppm is also a suitable alternative for replacing disinfectants based on glutaraldehyde [9,10]. A study of wastewater disinfection has shown that ClO_2_ is able to inactivate *E. coli*, although not as effective as chlorine of the same concentration [11].

Chlorine dioxide-based products have been created, which are designed to disinfect the air in the human breathing zone [12]. Chlorine dioxide is released when sodium chloride interacts with acid at high humidity. These products reduce the risk of getting infections in crowded places. ClO_2_ is a disinfectant that does not cause side effects due to its fast action and safe antimicrobial properties [7]. A recent review of dermatological reactions to various types of disinfectants used to reduce the risk of coronavirus infection has shown that ClO_2_ is safe to use and causes no damage to the skin [13,14]. ClO_2_ solution has also been closely studied for its potential use for the inactivation of viruses, including SARS-CoV-2, severe acute respiratory syndrome [15] and human papillomavirus (HPV) [16]. Interestingly, the ClO_2_ solution was also used to sterilize recycled respirators and surgical face masks during a critical shortage of such materials [6]. The study [17] showed that inactivation of the virus by treating it with low concentrations of chlorine dioxide prevents infection with the influenza virus by airborne transmission. The mechanism of inactivation of norovirus with ClO_2_ is achieved by the degradation of viral protein, including viral genomic RNA, and the disruption of the structure of the virus [18]. Treatment with ClO_2_ gas with a low concentration of about 0.05 ppm in an area with high humidity is useful for reducing the risk of human infection with norovirus without side effects [19].

Gaseous ClO_2_ has more advantages in disinfection compared to its solutions. Although aqueous chlorine dioxide ClO_2_ is used for food disinfection, its gaseous form is more effective for inactivating pathogenic microorganisms in inaccessible parts of products due to the high diffusion capacity and gas permeability. The corrosive activity and toxicity of chlorine dioxide gas are minimal in the concentrations used for disinfection; therefore, it is a more effective and generally accepted sterilizing agent [4,20].

ClO_2_ is stable in an aqueous solution, and in the gaseous state, ClO_2_ decomposes into chlorine and oxygen [21]. As a rule, chlorine dioxide is obtained from solutions of sodium chlorite or sodium chlorate as a result of their interaction with acids. If chlorine dioxide is used for water purification, chemical oxidation or disinfection, then sodium chlorite is used to obtain it, since in these cases, water that does not contain free chlorine is required [22]. The production of chlorine dioxide from sodium chlorite is much easier to control than the production of chlorine dioxide from sodium chlorate [23].

The most widely used method for determining chlorine dioxide is spectrophotometry [24]. The advantages of spectrophotometry are the following: ease of analysis, cost-effective equipment available in all laboratories, and the ability to automate the process. The method of chlorine dioxide determination in water using chlorophenol red is simple, sensitive, selective and has a range suitable for the determination of low concentrations of chlorine dioxide used in the treatment of drinking water. It is easy to reproduce the reaction of chlorophenol red with chlorine dioxide. However, no equation is currently postulated for the reaction [25].

The change in the color of the solution due to the reaction of chlorophenol red with chlorine dioxide follows the Beer–Lambert–Bouguer law at concentrations not higher than 1.0 mg L^−1^. It is noted that hypochlorous acid reacts with chlorine dioxide, but does not react with chlorophenol red [26].

The authors [27] made some additions to this method, including the use of a phosphate buffer (pH 7). Calibration lines were constructed for different standard buffers. The results showed that pH has a significant influence on the sensitivity of the technique and the linearity of the calibration line. The optimal pH value lies in the range of 7.0 to 7.5 units.

Thus, the validity of using chlorine dioxide as a disinfectant is quite obvious. In this paper, a phosphorylated woven fabric is proposed as a carrier matrix for sodium chlorite in view of its natural origin and the environmental friendliness of the material. In addition, unlike the previously proposed analogs, the selected material will allow using the disinfectant material repeatedly. After reducing the level of the released disinfectant to an ineffective concentration, the fabric can be subjected to special treatment and restore its bactericidal effect. The purpose of this work was to adapt the method of obtaining chlorine dioxide in concentrations suitable for air disinfection. The research objectives included the study of the solvents’ effect and air humidity on the concentration of chlorine dioxide. Such studies will optimize the operation of chlorine dioxide generators and understand the nature of the reaction that releases chlorine dioxide.

## 2. Materials and Methods

Phosphoric acid (85 wt. % in H_2_O), urea (99.0–100.5%), hydrochloric acid (37%), sodium thiosulfate (99.9%), iodine (≥99.99%), methyl alcohol (≥99.9%), ethyl alcohol (95.0%) and isopropyl alcohol (≥99.5%) used in this study were of reagent grade and purchased from Sigma Aldrich (St. Louis, MO, USA). Sodium chlorite (80%, pure) and chlorophenol red (pure) were also of reagent grade and acquired from Acros Organics (Waltham, MA, USA). All solutions were prepared in deionized, triply distilled water (specific resistance of 18.0 Ω m).

### 2.1. Cotton Cellulose Fabric Treatment

The commercially available mercerized cotton cellulose fabric with a fabric weight of 390 ± 9 g m^−2^, plain-weave yarn diameter of 250 ± 30 µm and fabric thickness of 700 ± 50 µm was used.

**Figure 1:**
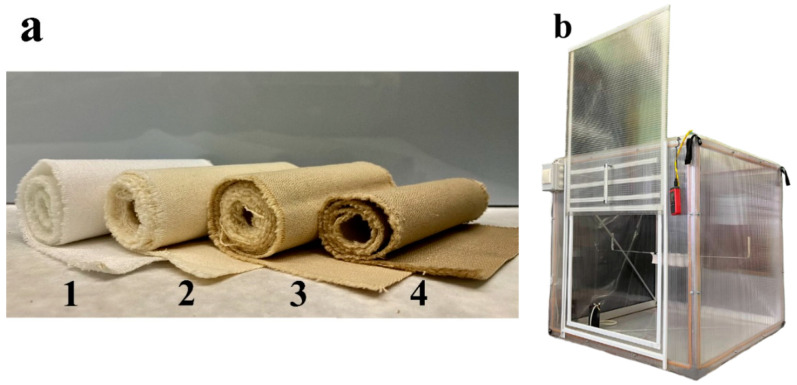
(**a**) Phosphorylated cotton cellulose fabric with different content of phosphoric acid in the phosphorylation mixture: 1—untreated fabric; 2—0 mol L^−1^; 3—0.80 mol L^−1^; 4—1.20 mol L^−1^. (**b**) Aspiration camera.

Fabric modification was carried out according to the procedure described in [28]. The dried fabric samples containing no more than 10% of moisture were treated with 2.80 mol L^−1^ urea solution, and 1.20, 0.80 or 0 mol L^−1^ phosphoric acid solutions at 80 °C for 1 h (the ratio of “sample” to “liquid” was 1:10). The treated samples were squeezed to a mass twice greater than the dry sample mass and dried at 70 °C for at least 5 h. After drying, the samples were heated at 145~150 °C for 1.5 h. After the phosphorylation process, the samples were washed three times with hot and cold distilled water and were treated with the 0.10 mol L^−1^ HCl solution at room temperature for 1 h. Then, the samples were washed three times with cold distilled water again and were dried at 70 °C for at least 5 h (Figure 1).

### 2.2. Phosphorus Content Determination

The phosphorus content in cotton fabric before and after phosphorylation was determined by the spectrophotometric method based on the color intensity of the Sn(II) reduced phosphorus molybdenum complex. Complete oxidation of phosphorus in the cellulose material samples into phosphate ions was carried out according to the method described in [28]. The optical density of the blue phosphor-molybdenum reduced complex was detected at a wavelength of 700 nm (a 5.00 cm thick cell was used). The experimental molar absorption coefficient for this complex was found to be equal to ε_700_ nm = (2.485 ± 0.023) × 10^4^ L mol^−1^ cm^−1^. According to the previously obtained calibration graph, the phosphorus content in the fabric was determined as mmol g^−1^ or mass percentages.

### 2.3. Tensile Strength

Tensile testing was performed on a Zwick Roell Z010 (Borken, Germany) setup. The width of the sample was shown to be 20 mm, and the length of fabric element being torn was 50 mm. Preload value was 0.10 N, and the stretching speed was 20 mm min^−1^. The standard maximum of the breaking force was measured until at least five reproducible results were obtained. The results were processed using *t*-test statistics.

### 2.4. Determination of the ClO_2_ Content in the Solution

A ClO_2_ solution with a concentration of approximately 0.8 mmol L^−1^ was prepared to plot the calibration curve and follow the determination of the ClO_2_ content in the air. This solution was kept in the dark at a temperature of 8 °C for 4 days to obtain a high stationary concentration of ClO_2_ in the solution. Iodometric titration with potentiometric indication was performed until at least five reproducible results were obtained to determine the exact concentration of ClO_2_ in the solution. The potentiometric titration of the samples was carried out through potentiometric titration with 16.0 mmol L^−1^ Na_2_SO_3_S using the voltmeter (“ECOTEST 120”, Econix, Moscow, Russia) (platinum electrode combined with silver chloride reference electrode).

### 2.5. Determination of the ClO_2_ Content in the Air

As a result of the reaction with chlorine dioxide, the solution of chlorophenol red discolored to white. The changes in the color of solutions were recorded on a spectrophotometer by the change in optical density at a wavelength of 571 nm in an optical cell with an optical path length of 5.00 cm. Different amounts of chlorine dioxide were added to the chlorophenol red solution. Calibration curve was plotted based on the obtained results.

The determination of the chlorine dioxide amount in the air was performed in an aspiration camera of nominal dimensions of 1 × 1 × 1 m and volume of 1.080 m^3^ (Figure 1b). It is made of cellular polycarbonate with a double-sided protective layer against ultraviolet light. The camera is equipped with two fans to homogenize the gas inside the camera, a humidity monitor and a thermometer to control humidity and temperature, respectively. The camera is connected in series with five modified Drexel absorbers filled with 10.00 ± 0.02 g of a working solution of chlorophenol red and a Kromschroeder BK-G 4 (Osnabrueck, Germany) volume meter of the passing gas for gas aspiration and absorption. Air was pumped out of the camera using an aspirator PU-4E (Moscow, Russia) for air sampling with an adjustable flow rate. The optimal aspiration rate was 5 L min^−1^. The volume of gas passed through the chlorophenol red solution varied, considering the amount of chlorine dioxide released. When chlorine dioxide passed through the absorbers, the reaction with chlorophenol red occurred; this resulted in discoloration of the solution to white. Optical densities of each absorber’s chlorophenol red solution were obtained using a spectrophotometer. The optical density of the last solution remained consonant with the initial solution. The obtained total changes in optical density of each absorber solution were correlated to the calibration curve, and the chlorine dioxide concentration in the aspiration camera was determined using it.

The measurements of optical spectra were carried out on a Cary 100 Scan UV–Visible Spectrophotometer (Santa Clara, CA, USA) equipped with a thermostatted cell holder. Cooling or heating of the cell was accomplished by built-in Peltier elements, which allowed variation of the temperature of the optical cell containing the solution. The optical spectra were measured at 20 °C.

### 2.6. Generation of ClO_2_

When sodium chlorite is applied to phosphorylated fabric, an antimicrobial agent, chlorine dioxide, is released due to interaction with active phosphorus-containing sites. Phosphorylated cotton fabric with a mass of 10 g was impregnated with a solution of NaClO_2_ until its mass increased by 3.00 ± 0.02 g. Then the fabric was placed in the center of the aspiration camera, the air was pumped out of it at regular intervals and later the concentration of chlorine dioxide was determined.

### 2.7. Temperature and Humidity in the Aspiration Camera

The temperature and humidity in the aspiration camera were measured by a digital thermohygrometer RGK TH-30 (Irkutsk, Russia). This device is equipped with a remote sensor on a long wire, which makes it convenient to carry out measurements inside the camera. The temperature measurement range is from −10 °C to +60 °C with an accuracy of ±0.5 °C. The humidity measurement range is from 10% to 95% with a reliability of readings from 3%.

## 3. Results and Discussion

The phosphorus content in the fabric for the concentration of phosphoric acid in the phosphorylating mixture of 0 mol L^−1^, 0.80 mol L^−1^ and 1.20 mol L^−1^ was 0.020 ± 0.002 mmol g^−1^, 0.320 ± 0.047 mmol g^−1^ and 0.802 ± 0.082 mmol g^−1^, respectively. Phosphorylation using a urea solution with a concentration of 2.80 mol L^−1^ and a phosphoric acid solution with a concentration of 1.20 mol L^−1^ allowed obtaining a material with an optimal ratio of phosphorus content and strength characteristics. The value of the tensile strength of the mercerized cellulose fabric was 17.2 ± 0.5 MPa. After the phosphorylation process, it decreases to 5.0 ± 1.0 MPa for any concentrations of phosphoric acid in the phosphorylating mixture. Phosphorylation takes place in a highly acidic environment and at a higher temperature. Therefore, during phosphorylation, there is a significant decrease in the strength characteristics of the fabric.

It was found that after 30 min of chlorine dioxide generation by samples of phosphorylated cellulose fabric with the amount of phosphorus equal to 0.802 ± 0.082 mmol g^−1^ and 0.320 ± 0.047 mmol g^−1^, an absolutely identical (within statistical error) ClO_2_ concentration of 1.43 ± 0.31 mg m^−3^ is detected in the aspiration camera. In our opinion, this is due to the fact that another component of the reaction—sodium chloride—is a limiting reagent. In this regard, with the same content of water and sodium chlorite in the impregnating solution, the formation of chlorine dioxide from the samples with different phosphorus content occurs in the same amount and at approximately equal rates. At the same time, for untreated fabric and a sample treated with a phosphoric acid-free mixture with a residual phosphorus content of 0.020 ± 0.002 mmol g^−1^, ClO_2_ release was not recorded.

Cotton-mercerized cellulose that was not subjected to the phosphorylation process does not contribute to the generation of chlorine dioxide. Cellulose fabric, which underwent all the processing procedures but in the absence of phosphoric acid, did not interact with the sodium chlorite solution. The stoichiometry and chemical mechanisms of the chlorine dioxide generation process are not completely clear. However, we assume that this occurs due to the interaction of sodium chlorite solution with phosphorus-containing groups. As it was noted earlier in [28], phosphorus-containing groups cannot be titrated in an aqueous solution, which indicates their extremely low acidic function. However, this does not prevent their reaction with NaClO_2_ and the release of ClO_2_ from the solution into the surrounding gas environment.

### Solvent Selection for Applying NaClO_2_ Solution

**Figure 2:**
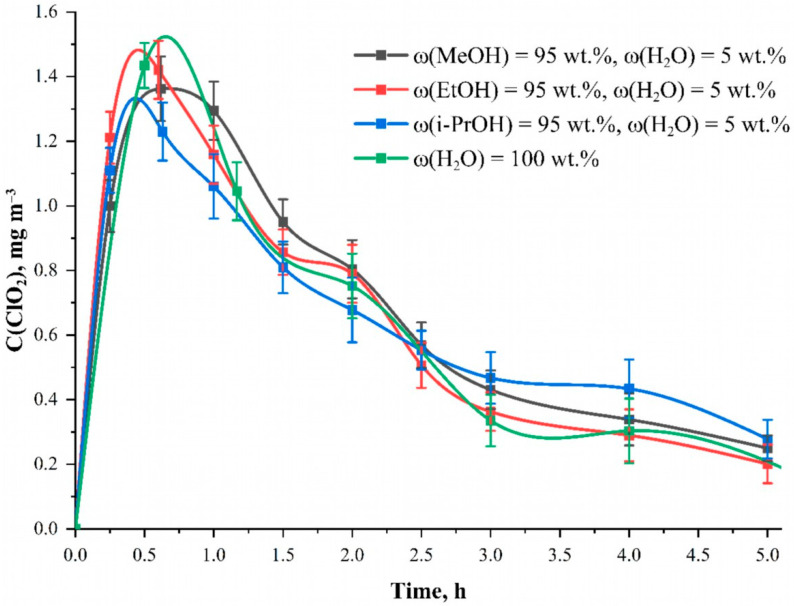
Dependencies of ClO_2_ concentration inside the camera when applying NaClO_2_ solution in different solvents onto the fabric surface at the time after the start of the reaction. Solution—C(NaClO_2_) = 25.0 mmol L^−1^, material—phosphorylated cotton cellulose fabric with the content of phosphorus-containing groups equal to 0.802 ± 0.082 mmol g^−1^.

Finely dispersed spray-up of sodium chlorite onto the fabric using an aerosol generator until the fabric mass increases by 3.00 ± 0.02 g was used as an optimal method of application. This method provides the most reproducible application of the main component to phosphorylated cellulose. In this case, the solvent must be able to wet out the material surface well. The cotton cellulose fabric has been pre-mercerized, which makes the surface hydrophilic and helps wet it out with water. Polar solvents such as distilled water, lower alcohols and acetone were chosen for the study due to their availability, widespread use in chemical practice, relative safety and cheapness. It was found that in pure methanol, ethanol, isopropanol and acetone, the solubility of sodium chlorite is low and instead of a real solution, a turbid sol of undissolved sodium chlorite particles and impurities in the solution was formed. Adding water to acetone did not achieve the desired effect and full dissolution did not occur. NaClO_2_ was successfully dissolved in water-alcohol solutions. The suspension was dissolved in an aliquot of distilled water prior to preparing solutions of sodium chlorite in alcohols. A solution containing 25.0 mmol L^−1^ of chlorite, 5 wt. % water and 95 wt. % of the corresponding alcohol was prepared. In most experiments, a sodium chlorite solution with a concentration of 25.0 mmol L^−1^ was used. Figure 2. presents the results of studies of the solvent effect on the release efficiency of chlorine dioxide over time.

**Figure 3:**
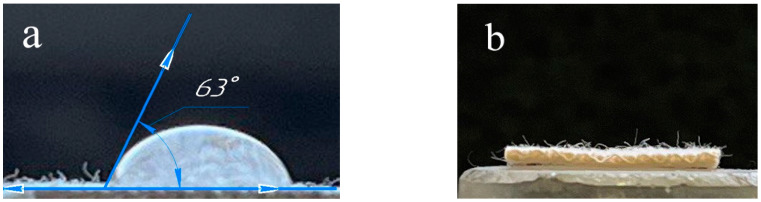
Photos of a sessile drop of (**a**) water and (**b**) the complete spreading of ethanol on the surface of phosphorylated cotton fabric.

The dependence of ClO_2_ formation at each moment is presented for four solvents: methanol, ethanol, isopropanol and water. It was found that the choice of solvent does not have a noticeable effect on the amount and nature of ClO_2_ release. The data for all solvents remains within the statistical error. Apparently, none of the alcohols participate in the reaction themselves but act solely as solvents. The advantage of alcohols was their high drying rate compared to water. Moreover, for all the alcohols contact angle was close to zero, which resulted in the instantaneous wet out of the phosphorylated cotton fabric, while for water, the contact angle value was equal to 63 ± 7° and the drop did not spread (Figure 3). This characteristic is of practical importance for the implementation of modified fabrics as chlorine dioxide generators. In further studies, ethanol was chosen due to its availability, safety and high evaporation rate.

**Figure 4:**
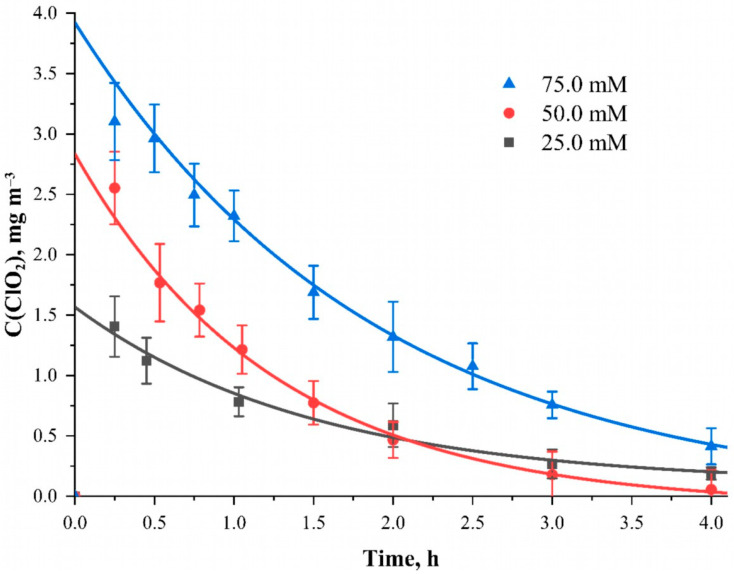
The dependence of the ClO_2_ concentration inside the camera when applying a NaClO_2_ solution of different concentrations to the material surface at the time after the start of the reaction. Solvent—95 wt. % water solution of ethanol; material—cotton cellulose fabric with the content of phosphorus-containing groups equal to 0.802 ± 0.082 mmol g^−1^.

The content of sodium chlorite in the ethanol solution varied in the range of 25~75 mmol L^−1^. The dependence of the chlorine dioxide generation at the indicated concentrations of sodium chlorite in the applied solutions is shown in Figure 4.

The above dependence shows that with an increase in the sodium chlorite content in the solution from 25.0 mmol L^−1^ to 75.0 mmol L^−1^, a proportional increase in the amount of the released ClO_2_ occurs. The maximum possible values of chlorine dioxide concentrations obtained by approximating its concentration to a time equal to 0 show values equal to 1.57, 2.84 and 3.92 mg m^–3^ for sodium chlorite concentrations in the impregnating solution with the concentration of 25.0, 50.0 and 75.0 mmol L^−1^, respectively. Plotting this dependence allowed us to obtain an approximate ratio between these concentrations. Taking into account the conditions of the experiment and other parameters and variables allows us to arrive at an approximate ratio of n(ClO_2_):n(ClO_2_^−^) = 0.296 ± 0.018. Presumably, the reaction of chloride ions with acid sites runs according to the following scheme:

4ClO_2_^−^ + 2H^+^ = 2ClO_2_ + ClO_3_^−^ + Cl^−^ + H_2_O

This shows that not all theoretically possible amounts of chlorine dioxide are released into the gas environment. In addition, the reaction may run differently, which will also affect the stoichiometric “reagent” to ”product” ratio.

For effective air disinfection with gaseous ClO_2_, the quantities formed at any of the concentrations presented are sufficient. However, a minimal and much more uniform decrease in concentration is observed when a solution with a concentration of 25.0 mmol L^−1^ is applied to cellulose fabric. In further experiments, a solution with a sodium chlorite content equal to 25.0 mmol L^−1^ was used for ease of determining chlorine dioxide amount in the air.

**Figure 5:**
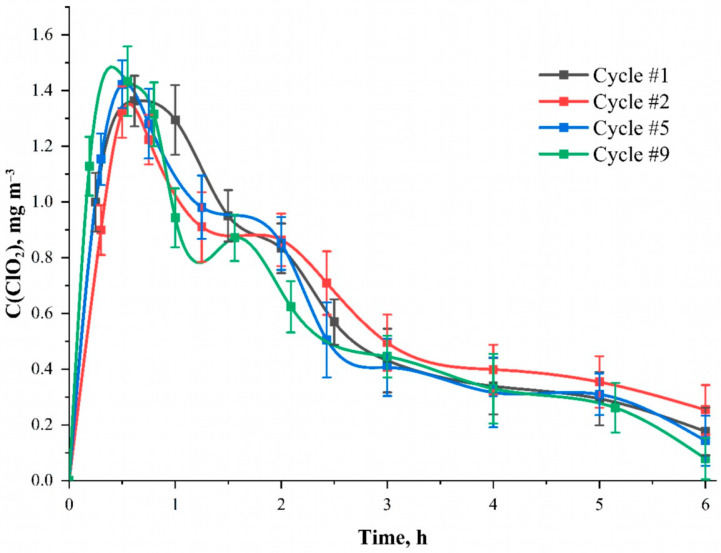
The dependence of the ClO_2_ concentration inside the camera during repeated application (cycle) of the NaClO2 solution to the material surface at the time after the start of the reaction. Solution—C(NaClO_2_) = 25.0 mmol L^−1^; solvent—95 wt. % aqueous ethanol solution; carrier—cotton cellulose fabric with the content of phosphorus-containing groups equal to 0.802 ± 0.082 mmol g^−1^.

The possibility of reusing the material or generator is also important. In the case of pulverizers and gas generators, this issue does not arise, since refueling them does not present any difficulty. However, for woven carrier materials, the number of usage cycles is an important characteristic. Figure 5 shows the dependence curves of the chlorine dioxide concentration inside the camera when the sodium chlorite solution is re-applied to the material surface at the time after the start of the reaction. Before each new cycle, the fabric was washed three times with cold and hot (80 °C) distilled water to remove the remnants of unreacted NaClO_2_ and then dried at 60 °C for 3 h in a drying oven. It was found that the number of application cycles does not affect the amount and nature of ClO_2_ formation in the gas phase. Such results indicate that the phosphorylated fabric acts not only as a chemical reagent but also as an active matrix, which initiates the process. Then, the possibility of using the modified cellulose is firstly limited to its strength characteristic and wear resistance. Thus, both components of the ClO_2_ generation reaction and its stoichiometry still remain objects for further research.

**Figure 6:**
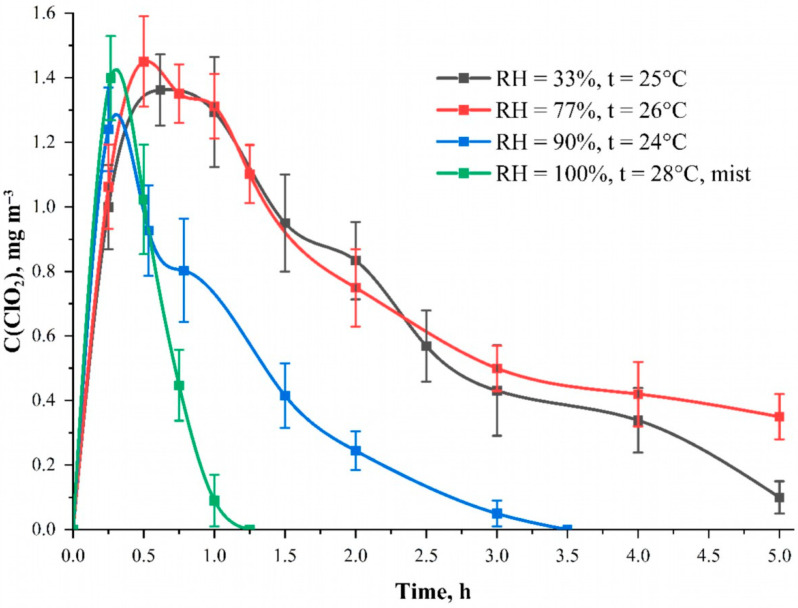
The dependence of the generated ClO_2_ concentration inside the aspiration camera on time at different air humidity. The curve “RH = 100%, t = 26 °C mist” corresponds to the humidity value of 100% checked by a hygrotermometer with visual observation of fog. Solution—C(NaClO_2_) = 25.0 mmol L^−1^; solvent—95 wt. % aqueous ethanol solution; carrier—cotton cellulose fabric with the content of phosphorus-containing groups equal to 0.802 ± 0.082 mmol g^−1^.

Individual protective means based on sodium chlorite and impregnated zeolites are being developed which are capable of releasing chlorine dioxide into the air in the presence of air moisture [29,30,31]. The influence of moisture in the gas phase of the aspiration camera on the formation and stability of ClO_2_ in the air was studied. Figure 6. shows the dependences of the generated chlorine dioxide concentration in the aspiration camera on time at different air humidity. The RH = 33% curve is responsible for the standard room humidity at an average temperature of 26 ± 2 °C. The results for other humidity parameters were obtained using an air humidifier. From the data obtained, the results show that an increase in humidity of 77% demonstrates the absence of humidity influence on the process of generation and consumption of ClO_2_ within the statistical error.

We assumed that the moisture in the air would be absorbed by the fabric and would initiate the reaction to chlorine dioxide formation. However, high humidity does not affect the process of chlorine dioxide generation from the fabric. Presumably, it is related to the presence of water in the solvent. We can assume that the water content has to be excessive for the reaction to occur. However, there is a sharp drop in the concentration of chlorine dioxide already in the first hour of measurements at 100% humidity in the presence of fog. This behavior, in our opinion, indicates that the water aerosol sorbs ClO_2_ molecules from the air and precipitates them from the gas phase. Thus, it can be assumed that the excessive moisture effectively removes the excess amount of ClO_2_ from the air space. This is one of the optimal ways to reduce the concentration of ClO_2_ to an approximately safe exposure level of about 0.1 mg m^–3^ [32].

It is known that chlorine dioxide decomposes under direct light. It has a fairly intense absorption band in the visible and ultraviolet regions of the spectrum [27,33,34,35,36]. Thus, the influence of xenon pulsed lamp radiation («Al’fa-05», Russia, Moscow) at the total flux intensity I_UV_ = 6.0 × 10^20^ quanta s^−1^ = 1.0 mE s^−1^ on chlorine dioxide in the gas phase was studied. To achieve this, a cloth soaked in a NaClO_2_ solution was placed inside the camera for 30 min. After 30 min of chlorine dioxide generation in each experiment, the gas phase was irradiated with ultraviolet light from a pulsed xenon lamp. ClO_2_ concentrations in the aspiration camera were determined before and after irradiation. It was found that chlorine dioxide completely decomposes in 15 min of irradiation (Table 1). Although irradiation for a shorter time causes degradation of chlorine dioxide, it nonetheless does not lead to the complete decomposition of ClO_2_.

The pulsed xenon lamp used by us was a powerful source of ultraviolet light. Complete decomposition of the same amount of chlorine dioxide in an aqueous solution as in the gas phase under UV irradiation of this lamp occurred within 27 s. However, experimental data show that the decomposition of chlorine dioxide in the gas phase is much slower, although it still occurs when its aqueous solution is irradiated with UV light [37,38,39,40]. On this account, the influence of daylight and sunlight on the decomposition of chlorine dioxide in the air can be neglected. Using this method of removing chlorine dioxide residues from the air after disinfection is possible, although it requires more time.

As represented by the conducted studies, the influence of various factors on chlorine dioxide, its concentration and stability in the air, and the main time of its generation from a fabric carrier was established. To achieve this, after permeating the fabric with aqueous ethanol solution NaClO_2,_ it was placed in an aspiration camera for 5 min after a certain waiting time and the concentration of ClO_2_ in the air was measured. It was found that 0, 10, 20, 30 and 40 min of waiting outside the aspiration camera, the bactericidal material releases ClO_2_ in concentrations of 0.62 ± 0.13, 0.35 ± 0.08, 0.11 ± 0.03, 0.0088 ± 0.0019 and 0 mg m^–3^, respectively. It means that the main generation of ClO_2_ occurs within the first 25–35 min. A further decrease in the ClO_2_ concentration corresponds only to the decomposition of ClO_2_ that was formed during the first 25–35 min of the reaction.

## 4. Conclusions

As a rule, long-acting chlorine dioxide generators operate on the principle of a chemical reaction occurring inside or on the surface of the precursor-impregnated matrix. In this paper, a new method of application is proposed: impregnation of the cellulose matrix with a sodium chlorite solution sprayed by an aerosol generator. Such a fabric is able to withstand repeated use, which was shown in this work. Lower alcohols (methanol, ethanol and isopropanol) are suggested as optimal polar solvents for the application of the solution to phosphorylated cotton cellulose fabric. The best ratio between the solubility of sodium chlorite, wetting properties and the high evaporation rate of the solvent itself is observed when using 95% ethanol. It is shown that the generation of chlorine dioxide by cellulose fabric impregnated with such a solution occurs within the first 25–35 min. Further, only a decrease in the concentration accumulated during this time occurs due to absorption, sedimentation, and degradation.

It has been established that humidity does not improve or accelerate the process of chlorine dioxide release in any way. Moreover, the high moisture content and especially fog in the air causes the fast and complete absorption of ClO_2_ by microdrops and its removal from the gas environment. Understanding these processes is important because the use of chlorine dioxide in the presence of a person is life-threatening and prohibited.

After irradiating the room air with a lamp emitting ultraviolet light, the concentration of ClO_2_ decreased significantly. However, the rate of chlorine dioxide decomposition in the gas phase at low humidity is less than the rate of chlorine dioxide decomposition in an aqueous solution, where it occurs in a few seconds. This suggests that the most promising way to remove chlorine dioxide from the air after disinfection is a combined method: irradiation of rooms with high humidity with ultraviolet light.

## Figures and Tables

**Table 1:** Investigation of the effect of xenon pulsed lamp radiation on chlorine dioxide in the gas phase.

№	Time of Irradiation of the Gas Phase with UV Light of a Pulsed Xenon Lamp, Min	C(ClO_2_), mg m^–3^
1	0	1.70 ± 0.21
2	5	0.365 ± 0.059
3	10	0.155 ± 0.028
4	15	0

## Data Availability

Not applicable.
